# Utility of Urinary β2-Microglobulin for Detection of Renal Sarcoidosis Without Pulmonary Involvement: A Case Report

**DOI:** 10.3390/reports9010082

**Published:** 2026-03-10

**Authors:** Yuri Oue, Ryosuke Saiki, Tomohiro Murata, Kan Katayama, Kaoru Dohi

**Affiliations:** Department of Cardiology and Nephrology, Graduate School of Medicine, Mie University, Tsu 514-8507, Japan

**Keywords:** sarcoidosis, β2-microglobulin, interstitial nephritis, tubular markers

## Abstract

**Background and Clinical Significance:** Sarcoidosis is a systemic inflammatory disorder characterized by noncaseating granulomas. While pulmonary involvement is common, isolated renal involvement is rare and diagnostically challenging. We report a case emphasizing the utility of urinary tubular markers for early detection. **Case Presentation:** A 60-year-old woman with a history of suspected ocular sarcoidosis presented with progressive renal impairment and constitutional symptoms. Initial workup for systemic sarcoidosis was negative, leading to a misdiagnosis of chronic fatigue syndrome. Her rising serum creatinine was initially attributed to dehydration. However, a marked elevation in urinary β2-microglobulin (33,736 μg/L) prompted a renal biopsy, which revealed granulomatous tubulointerstitial nephritis. Following prednisolone therapy, her renal function improved, and her fatigue resolved completely. **Conclusions:** This case demonstrates that the kidney can be the primary site for histological diagnosis in the absence of pulmonary lesions. Incorporating urinary β2-microglobulin into routine monitoring may facilitate the early detection of renal sarcoidosis, preventing diagnostic delays.

## 1. Introduction and Clinical Significance

Sarcoidosis is a systemic inflammatory disorder of unknown etiology, characterized by the presence of noncaseating granulomas [1]. Although pulmonary involvement is the most common manifestation, the disease can affect virtually any organ, including the skin, eyes, heart, nervous system, and kidneys [2]. Renal involvement is relatively rare, occurring in approximately 6% of patients with sarcoidosis [3]. The diagnosis of sarcoidosis requires compatible clinical manifestations and histological evidence of noncaseating granulomas, which makes it challenging to establish a definitive diagnosis when tissue sampling is not feasible [4].

When sarcoidosis is strongly suspected but tissue sampling is not feasible, regular follow-up at approximately 6-month intervals is recommended [5]. Even after a diagnosis is established, continuous monitoring is essential for the early detection of new organ involvement [4,6]. Currently, there is no internationally standardized protocol regarding the optimal follow-up interval for patients diagnosed with sarcoidosis. Guidelines recommend annual measurements of serum creatinine and serum calcium as part of renal function monitoring in these patients [4]. In addition, previous reports have suggested follow-up intervals of every 3–6 months during the first 2 years after diagnosis, followed by annual monitoring for the subsequent 3–5 years [6].

We report a case of sarcoidosis diagnosed by kidney biopsy performed for subsequent renal impairment, as the patient initially lacked biopsy-accessible lesions. The renal dysfunction was initially attributed to a prerenal cause due to anorexia, given the scarcity of proteinuria and hematuria. This case suggests that adding tubular markers to follow-up monitoring may facilitate early diagnosis.

## 2. Case Presentation

A 60-year-old woman was admitted to our nephrology department for the evaluation of progressive renal involvement over a 7-month period. Her past medical history included type 2 diabetes mellitus, managed with linagliptin, and hypertension. One year prior to admission, the patient presented with bilateral blurred vision, and sarcoid uveitis was suspected based on findings of bilateral angle nodules, peripheral anterior synechiae, vitreous “snowball” opacities, and chorioretinal atrophic lesions. The uveitis responded well to topical corticosteroid treatment.

Several months later, she began to experience constitutional symptoms, including fatigue, intermittent fever, anorexia, and a 5 kg weight loss. Systemic sarcoidosis was initially suspected, prompting workups for pulmonary, cardiac involvement, and other organ involvement. However, a computed tomography scan did not reveal hilar lymphadenopathy or nephrocalcinosis, and cardiac evaluation including echocardiography and electrocardiography, was unremarkable. Consequently, a diagnosis of chronic fatigue syndrome was made. However, her symptoms persisted. Concurrently, serum creatinine levels gradually rose from a normal baseline to 0.86 mg/dL and then to 1.30 mg/dL. Although this impairment was initially attributed to a prerenal cause secondary to anorexia given the absence of proteinuria and hematuria, the continued rise in creatinine to 1.47 mg/dL prompted a referral to our nephrology department.

**Figure 1:**
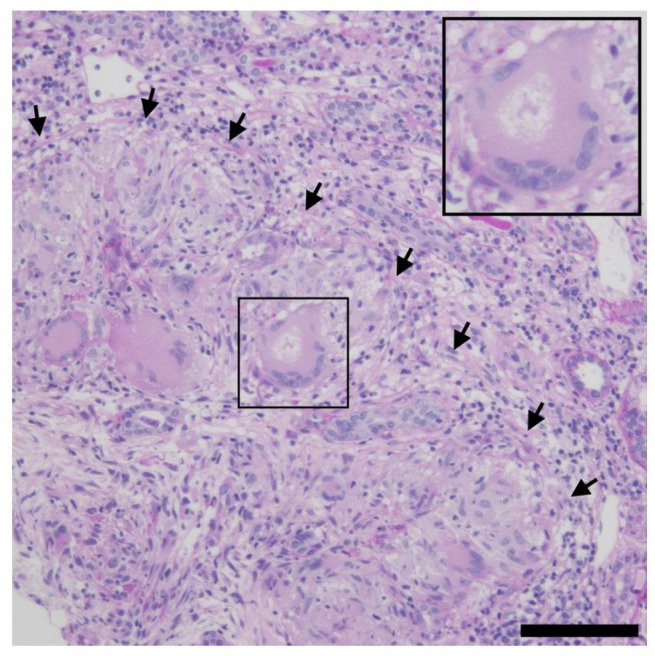
Light microscopy (Interstitium). Kidney biopsy revealed tubulointerstitial nephritis with multiple noncaseating granulomas (arrow) containing numerous multinucleated giant cells and an asteroid body. An asteroid body is shown in the boxed area and magnified in the inset. Bar = 100 μm.

**Figure 2:**
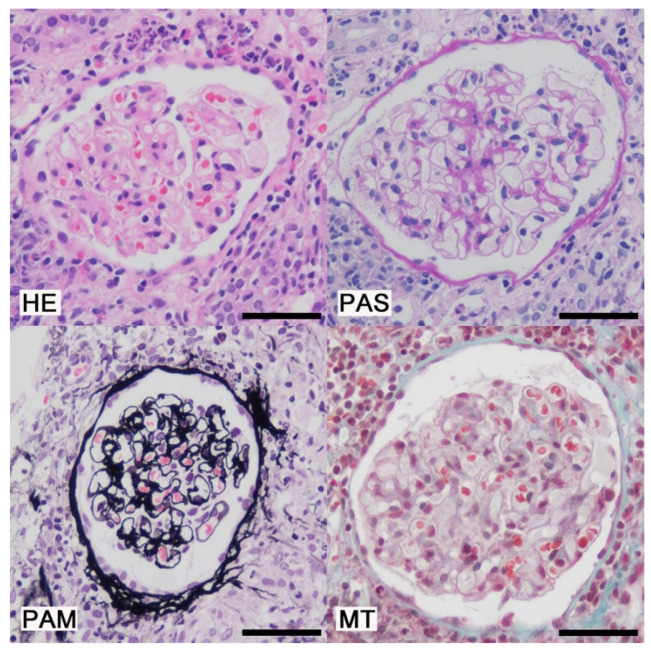
Light microscopy (Glomerulus). Hematoxylin and Eosin (HE) staining showed no signs of inflammatory cell infiltration in the glomeruli. Periodic acid-Schiff (PAS) staining showed no signs of mesangial proliferation, crescents, or adhesion. Periodic acid methenamine silver (PAM) staining showed no thickening, duplication, or spikes in the glomerular basement membrane. Masson-Trichrome (MT) staining showed no signs of immune complex deposits in the glomeruli. Bars = 50 μm.

**Figure 3:**
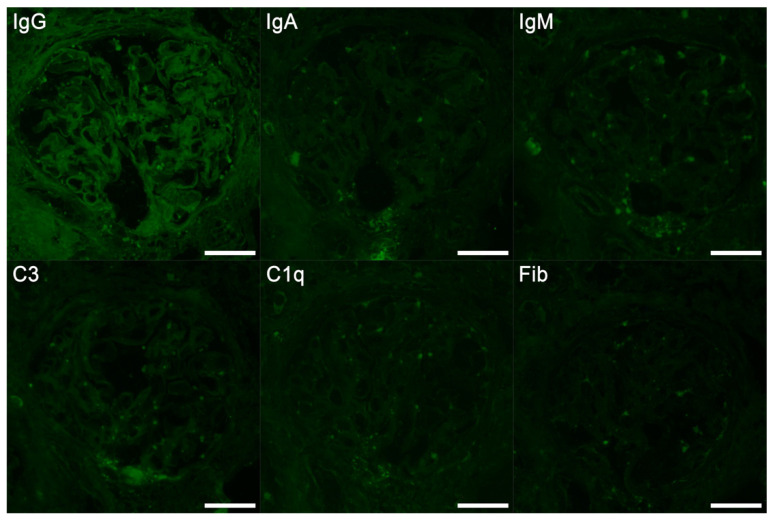
Immunofluorescence study. Immunofluorescence showed no staining. Bars = 50 μm.

**Figure 4:**
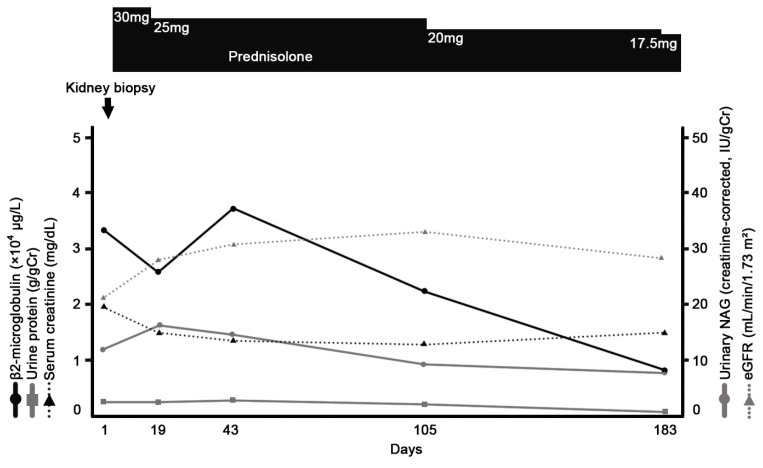
The clinical course. Abbreviations: eGFR, estimated glomerular filtration rate; NAG, N-acetyl-β-D-glucosaminidase; IU, international unit.

On admission, her blood pressure was 138/84 mmHg, and pulse was 89 beats/min. She had intermittent fevers of around 38.5 °C every 2–3 days. She had a normal body mass index of 19.0 kg/m^2^ (height 1.67 m and weight 53 kg). Physical examination revealed no other abnormalities. Laboratory data are listed in Table 1. Although urinalysis showed no significant proteinuria or hematuria, key results included an elevated serum creatinine level of 1.97 mg/dL, and markedly elevated urinary levels of β2-microglobulin (33,736 μg/L) and N-acetyl-β-D-glucosaminidase (12.1 U/gCr), suggesting tubulointerstitial injury. Spot urine was used for the analysis. Although the urine pH was not adjusted prior to testing, the patient’s urine pH at presentation was 6.0, and it ranged between 6.0 and 7.5 throughout the clinical course, minimizing the risk of β2-microglobulin degradation. Urinary β2-microglobulin was measured via latex agglutination turbidimetric immunoassay using the BMG-Latex X1 “Seiken” kit (Denka Company Limited, Tokyo, Japan; institutional reference range, 0–150 μg/L). Serum calcium and angiotensin-converting enzyme levels were within normal limits, whereas the soluble interleukin-2 receptor level was elevated at 1108 U/mL. Serological markers for autoimmune diseases were negative. A renal biopsy revealed tubulointerstitial nephritis with multiple noncaseating granulomas containing numerous multinucleated giant cells and an asteroid body (Figure 1). The glomeruli appeared normal (Figure 2), and immunofluorescence studies were negative for immunoglobulins and complement components (Figure 3). To exclude other etiologies of granulomatous interstitial nephritis, comprehensive serological testing was performed. The interferon-γ release assay, antineutrophil cytoplasmic antibodies, antinuclear antibodies, and anti–Sjögren’s syndrome-related antigens were all negative. Acid-fast bacilli stains, fungal stains, polymerase chain reaction for Mycobacterium tuberculosis, and tissue cultures were not performed on the biopsy specimen; however, tuberculosis was considered clinically unlikely due to the negative interferon-γ release assay result. Although serum IgG4 levels were not measured and IgG4 immunostaining was not performed on the biopsy specimen, IgG4-related disease was considered unlikely because the serum IgG level was within the normal range, and the biopsy specimen lacked the characteristic storiform fibrosis of IgG4-related disease. Furthermore, drug-induced tubulointerstitial nephritis caused by linagliptin was considered unlikely, given the absence of reported cases implicating this agent. Finally, tubulointerstitial nephritis and uveitis (TINU) syndrome was considered but excluded based on the specific ocular manifestations. While TINU typically manifests as non-granulomatous anterior uveitis, this patient presented with distinct features of granulomatous panuveitis, which is characteristic of ocular sarcoidosis. Specifically, the presence of bilateral angle nodules indicates a granulomatous process, and the posterior segment involvement evidenced by vitreous “snowball” opacities and chorioretinal atrophic lesions strongly supports a diagnosis of sarcoidosis over TINU, as the latter is predominantly an anterior segment disorder. Based on these findings, a definitive diagnosis of renal sarcoidosis was established. She was treated with oral prednisolone (30 mg/day). Treatment improved her renal function, with decreasing serum creatinine and urinary β2-microglobulin levels (Figure 4). Her intermittent fevers and fatigue resolved, and her vision improved further. The prednisolone dose was tapered to 25 mg/day after 2 weeks, and she was discharged. Six months after the initiation of treatment, the prednisolone dose had been tapered to 17.5 mg/day, with her serum creatinine partially improved to 1.50 mg/dL and her urinary β2-microglobulin level decreased to 8346 μg/L. Although hyperglycemia was noted following corticosteroid initiation, it was managed by adjusting her anti-diabetic medication without severe adverse events. We plan to continue gradually tapering the prednisolone.

## 3. Discussion

This case highlights two lessons: (i) the kidney can be the first organ amenable to biopsy in sarcoidosis, and (ii) in addition to regular blood tests, monitoring tubular markers can contribute to early detection.

More than 90% of patients with sarcoidosis and extrarenal lesions have pulmonary involvement [7], so the absence of lung lesions makes diagnosis difficult. Renal sarcoidosis usually manifests as nephrocalcinosis associated with hypercalcemia or as granulomatous interstitial nephritis [8]. Acute kidney injury with hypercalcemia strongly suggests renal sarcoidosis [9], whereas granulomatous interstitial nephritis without hypercalcemia is harder to recognize. In such cases, urinalysis often shows only minimal proteinuria or hematuria, and rising serum creatinine may be the sole abnormality on routine testing. However, the biological variation in serum creatinine is approximately 4.4–4.7% [10], so early, mild increases may be interpreted as normal fluctuation. As in our case, when there is an episode suggestive of dehydration, creatinine elevation is easily attributed to a prerenal cause, and patients are often managed only with encouragement of fluid intake and observation. Although the frequency is low, clinicians should always consider that sarcoidosis can be complicated by renal involvement.

In addition to regular blood tests, monitoring tubular markers can contribute to early detection of renal involvement. Due to the previously mentioned biological variation in creatinine, a change of 10–15% is necessary to determine a significant increase [10]. In practice, small changes in serum creatinine are difficult to interpret and are frequently attributed to prerenal factors such as dehydration, especially in patients with anorexia. Halbritter et al. recently warned against such “diagnoses of convenience”. They emphasized that this tendency leads to misclassification or delayed diagnosis particularly in cases with bland urinary sediment [11]. To avoid overlooking treatable etiologies under the guise of “dehydration,” objective evidence is required to justify invasive procedures like renal biopsy. Interstitial nephritis elevates tubular injury markers, which tend to increase earlier than serum creatinine [12,13,14]. In the present case, urinary β2-microglobulin was disproportionately elevated compared to serum creatinine. This is because serum creatinine reflects a decline in glomerular filtration rate, whereas tubular markers directly reflect tubular injury and inflammation, often rising before overt renal involvement becomes apparent [14]. Therefore, we hypothesize that incorporating tubular markers in routine follow-up might facilitate the detection of renal involvement in sarcoidosis patients and could serve as an additional clue for considering a renal biopsy.

This report has several limitations. First, as this is a single case report, our observations are hypothesis-generating, and larger longitudinal studies are needed to confirm the utility of monitoring tubular markers in sarcoidosis. Second, urinary β2-microglobulin is a nonspecific marker of tubular injury and should be interpreted alongside clinical, imaging, and histopathological data. It can be elevated in various other causes of tubulointerstitial nephritis, and it may remain normal if pure glomerular disease is present, yielding false negatives. Finally, while the urine pH in our patient remained between 6.0 and 7.5, β2-microglobulin is known to degrade in highly acidic urine. Without strict pH control or alkalinization, there is a potential risk of underestimating β2-microglobulin levels in general practice.

## 4. Conclusions

Renal biopsy may be crucial for establishing the diagnosis of sarcoidosis in patients without pulmonary involvement. While our findings are based on a single case, routine incorporation of tubular markers might aid in the earlier recognition of renal sarcoidosis, although further studies are required to validate this approach.

## Figures and Tables

**Table 1:** **Laboratory data upon admission in the nephrology** **ward.**

**Urinary Examination**		**Blood Chemistry**	** **
pH (4.5–7.5)	6	TP (g/dL, 6.6–8.1)	6.9
Protein (g/gCr)	0.28	Alb (g/dL, 4.1–5.1)	3.9
Occult blood	(-)	BUN (mg/dL, 8–20)	18.2
		Cr (mg/dL, 0.46–0.79)	1.97
β2MG (μg/L, 0–150)	33,648	eGFR (mL/min/1.73 m^2^)	21.1
NAG (U/L, 0–11.5)	4.3	UA (mg/dL, 2.6–5.5)	4.1
NAG/Cr (U/gCr, 0–5.6)	12.1	Na (mEq/L, 138–145)	134
BJP	(-)	K (mEq/L, 3.6–4.8)	3.7
		Cl (mEq/L, 101–108)	99
Complete blood count		Ca (mg/dL, 8.8–10.1)	9.5
WBC (/μL, 3300–8600)	4500	P (mg/dL, 2.7–4.6)	3.5
RBC (×10^4^/μL, 435–555)	358	AST (U/L, 13–30)	9
Hb (g/dL, 13.7–16.8)	10.2	ALT (U/L, 7–23)	8
Plt (×10^4^/μL, 15.8–34.8)	24.5	LDH (U/L, 124–222)	161
		CRP (mg/dL, 0–0.14)	2.03
Serology			
ANA	1:80	IgG (mg/dL, 861–1747)	1386
Homogeneous pattern	1:80	IgA (mg/dL, 93–393)	215
Speckled pattern	1:80	IgM (mg/dL, 33–183)	80
Anti-SSA (U/mL, 0–7.0)	0.4	CH50 (U/mL, 31.6–57.6)	53.9
Anti-SSB (U/mL, 0–7.0)	0.6	sIL-2R (U/mL, 122–496)	1108
Anti-dsDNA (IU/mL, 0–10.0)	3.7	lysozyme (μg/mL 5.0–10.2)	13.3
TB IFNγ	(-)	ACE (U/L, 8.3–21.4)	17.3

Alb, albumin; ALT, alanine transaminase; ANA, antinuclear antibody; Anti-dsDNA, anti-double-stranded DNA antibody; AST, asparate transaminase; BJP, Bence Jones protein; BUN, blood urea nitrogen; Ca, calcium; CH50, 50% hemolytic complement activity; Cl, chloride; Cr, creatinine; CRP, C-reactive protein; eGFR, estimated glomerular filtration rate; Hb, hemoglobin; IgA; immunoglobulinA, IgG, immunoglobulin G; IgM; immunoglobulin M; K, kalium; LDH, lactate dehydrogenase; Na, natrium; NAG, N-acetyl-β-D-glucosaminidase; Plt, platelets; RBC, red blood cells; TB IFNγ, QuantiFERON-Plus TB IFNγ; TP, total protein; UA, uric acid; WBC, white blood cells.

## Data Availability

The original data presented in this study are available on reasonable request from the corresponding author. The data are not publicly available due to privacy concerns.
